# Impact of Global Geographic Region on Time in Therapeutic Range on Warfarin Anticoagulant Therapy: Data From the ROCKET AF Clinical Trial

**DOI:** 10.1161/JAHA.112.000067

**Published:** 2013-02-22

**Authors:** Daniel E. Singer, Anne S. Hellkamp, Jonathan P. Piccini, Kenneth W. Mahaffey, Yuliya Lokhnygina, Guohua Pan, Jonathan L. Halperin, Richard C. Becker, Günter Breithardt, Graeme J. Hankey, Werner Hacke, Christopher C. Nessel, Manesh R. Patel, Robert M. Califf, Keith A. A. Fox, ROCKET AF Investigators

**Affiliations:** 1 Department of Medicine, Massachusetts General Hospital and Harvard Medical School, Boston, MA (D.E.S.); 2 Duke Clinical Research Institute, Duke University Medical Center, Durham, NC (A.S.H., J.P.P., K.W.M., Y.L., R.C.B., M.R.P.); 3 Division of Cardiology, Department of Medicine, Duke University Medical Center, Durham, NC (J.P.P., K.W.M., R.C.B., M.R.P., R.M.C.); 4 Johnson & Johnson Pharmaceutical Research and Development, Raritan, NJ (G.P., C.C.N.); 5 The Cardiovascular Institute, Mount Sinai School of Medicine, New York, NY (J.L.H.); 6 Department of Cardiology and Angiology, Hospital of the University of Münster, Münster, Germany (B.); 7 Stroke Unit, Department of Neurology, Royal Perth Hospital, Perth, Australia (G.J.H.); 8 Department of Neurology, Ruprecht‐Karls‐University, Heidelberg, Germany (W.H.); 9 Duke Translational Medicine Institute,; 10 University of Edinburgh and Royal Infirmary of Edinburgh, Edinburgh, UK (K.A.F.)

**Keywords:** anticoagulants, arrhythmia, embolism, prevention, risk factors

## Abstract

**Background:**

Vitamin K antagonist (VKA) therapy remains the most common method of stroke prevention in patients with atrial fibrillation. Time in therapeutic range (TTR) is a widely cited measure of the quality of VKA therapy. We sought to identify factors associated with TTR in a large, international clinical trial.

**Methods and Results:**

TTR (international normalized ratio [INR] 2.0 to 3.0) was determined using standard linear interpolation in patients randomized to warfarin in the ROCKET AF trial. Factors associated with TTR at the individual patient level (i‐TTR) were determined via multivariable linear regression. Among 6983 patients taking warfarin, recruited from 45 countries grouped into 7 regions, the mean i‐TTR was 55.2% (SD 21.3%) and the median i‐TTR was 57.9% (interquartile range 43.0% to 70.6%). The mean time with INR <2 was 29.1% and the mean time with an INR >3 was 15.7%. While multiple clinical features were associated with i‐TTR, dominant determinants were previous warfarin use (mean i‐TTR of 61.1% for warfarin‐experienced versus 47.4% in VKA‐naïve patients) and geographic region where patients were managed (mean i‐TTR varied from 64.1% to 35.9%). These effects persisted in multivariable analysis. Regions with the lowest i‐TTRs had INR distributions shifted toward lower INR values and had longer inter‐INR test intervals.

**Conclusions:**

Independent of patient clinical features, the regional location of medical care is a dominant determinant of variation in i‐TTR in global studies of warfarin. Regional differences in mean i‐TTR are heavily influenced by subtherapeutic INR values and are associated with reduced frequency of INR testing.

**Clinical Trial Registration:**

URL: ClinicalTrials.gov. Unique identifier: NCT00403767.

## Introduction

Both the efficacy and safety of warfarin anticoagulation in patients with atrial fibrillation (AF) are strongly dependent on the intensity of anticoagulation measured as the international normalized ratio (INR). The risk of ischemic stroke increases with INR levels <1.8, and the risk of intracranial hemorrhage increases sharply at INR levels >3.5.^1^ These findings support the standard “therapeutic” INR range of 2.0 to 3.0 for atrial fibrillation.^2–4^ A commonly used summary of the quality of warfarin anticoagulation is the linearly interpolated percent time in the therapeutic range (TTR).^5–7^ While many patient‐ and system‐level variables have been demonstrated to affect the INR, and there have been analyses of variation of average TTR at the institutional or geographic level,^8–9^ there are relatively few large studies assessing the impact of patient features on TTR at the level of the individual patient.^10^ In the current study, we explored individual and regional determinants of TTR among patients randomly allocated to warfarin in the global ROCKET AF (Rivaroxaban Once daily oral direct factor Xa inhibition Compared with vitamin K antagonism for prevention of stroke and Embolism Trial in Atrial Fibrillation) double‐blind trial of rivaroxaban versus adjusted‐dose warfarin in patients with atrial fibrillation.^11–12^

## Methods

The design, conduct, and main results of the ROCKET AF trial have been presented previously.^11–12^ In brief, rivaroxaban (20 mg daily; 15 mg daily in patients with creatinine clearance of 30 to 49 mL/min) was compared with adjusted‐dose warfarin (INR point target of 2.5, INR range 2.0 to 3.0) for the prevention of stroke or systemic embolism. Patients with electrocardiographically documented nonvalvular atrial fibrillation at moderate to high risk of stroke were recruited at 1178 participating sites in 45 countries. Elevated risk was indicated by a history of stroke, transient ischemic attack (TIA), or systemic embolism or ≥2 of the following: heart failure or left ventricular ejection fraction ≤35%, hypertension, age ≥75 years, or diabetes mellitus (CHADS_2_ score ≥2).^13^ The proportion of patients without prior ischemic stroke, TIA, or systemic embolism and ≤2 risk factors was limited to 10% of the cohort by region; the remainder required either prior thromboembolism or ≥3 risk factors. Investigators were chosen on the basis of performance in clinical trials and access to large clinical practices that included patients with atrial fibrillation. We do not have comprehensive information on recruiting physicians' specialty status. Warfarin dosing was managed by local physicians based on INR values generated by a standard point‐of‐care device (HemoSense, San Jose, CA). While physicians were reminded about the INR target of the trial and the need for monthly INR tests even when patients' anticoagulation status was stable,^14^ the study did not provide specific treatment algorithms for anticoagulation management. Patients with <6 weeks of exposure to vitamin K antagonist (VKA) medication immediately before entry into the trial were considered VKA naïve.

### Statistical Analysis

For the current analyses, patients were included if they had been assigned to warfarin in the ROCKET AF trial and took ≥1 dose of warfarin and had ≥1 INR test. Daily INR values between tests were imputed using the Rosendaal technique,^6^ and individual patient‐level TTR (i‐TTR) was calculated as the proportion of daily values within a strict range of INR 2.0 to 3.0. This included time during the initiation of warfarin at the start of the trial and after temporary interruptions but did not include time during temporary interruptions of ≥7 days or any time after permanent discontinuation. Only 0.18% of inter‐INR test intervals were >8 weeks. Univariable relationships between baseline variables and i‐TTR were assessed with single‐predictor linear regression models. A multivariable model was developed using multiple linear regression in which a set of independent predictors was chosen in stepwise fashion from a set of candidate predictors. These candidates were age, sex, geographic region, body mass index, systolic and diastolic blood pressures, atrial fibrillation type, hypertension, diabetes, prior stroke or TIA, coronary artery disease, chronic obstructive pulmonary disease, peripheral artery disease, prior gastrointestinal bleed, liver disease, alcohol consumption in the past 12 months, CHADS_2_ score, estimated glomerular filtration rate (Modification of Diet in Renal Disease equation),^15^ hemoglobin, patient medications, and type of prior VKA experience. Only variable values at entry to the study were used. Multivariable models were developed both with and without a random effect for center; we report only the results for the models without a random effect for center, because both modeling approaches produced highly similar results. Regional and country mean i‐TTRs were unweighted averages of i‐TTR values for all individuals within the given region or country, respectively.

The relationship between geographic region and i‐TTR was further characterized using linear regression models with region as the only predictor within subgroups defined by prior VKA experience, both for i‐TTR and for i‐TTR excluding the first 90 days of warfarin therapy. For the current analysis, we grouped the countries involved in the ROCKET AF trial into the following regions: East Asia (China, Hong Kong, Korea, Malaysia, Philippines, Singapore, Thailand, and Taiwan); India; Eastern Europe (Bulgaria, Czech Republic, Greece, Hungary, Lithuania, Poland, Romania, Russia, Turkey, and Ukraine); Western Europe and similar (Western Europe/similar: Australia, Austria, Belgium, Switzerland, Germany, Denmark, Spain, Finland, France, Great Britain, Israel, Italy, Netherlands, Norway, New Zealand, and Sweden); South Africa; Latin America (Argentina, Brazil, Chile, Colombia, Mexico, Peru, and Venezuela); and Canada/United States. These regional groupings were modified from those used in the primary trial report to provide more cultural and ethnic homogeneity.^12^ Analyses done with the original regional groupings reproduced the same patterns of regional effect on average i‐TTR although the overall *R*^2^ values for the multivariable models were modestly reduced (data not shown). We did not include terms for both region and race in the same multivariable models because the 2 were almost completely collinear. We summarize stroke risk using the CHADS_2_ score.^13^ Statistical significance of differences in the width of distributions of INR values across regions was assessed using the Miller jackknife technique.^16^

### Human Subjects

All individuals enrolled in the study gave informed consent. All appropriate national regulatory authorities and ethics committees at participating centers approved the study. An international executive committee designed the study and takes responsibility for the accuracy and completeness of all analyses.

## Results

### Baseline Patient Features

The ROCKET AF trial recruited atrial fibrillation patients at high risk for ischemic stroke. In the subpopulation (n=6983) included in the current analysis, the mean age was 71 years (median 73 years), 61% were male, 52% had had a prior stroke or TIA, and the mean CHADS_2_ stroke risk score was 3.3. A total of 83% were white, 12% were Asian, and there was a small representation of other racial/ethnic groups. Enrolled patients came from a broad set of geographic regions: 38% from Eastern Europe, 19% from Canada/United States, 16% from Western Europe/similar, 13% from Latin America, and 10% from East Asia (Table****1). Thirty‐seven percent of patients were VKA naïve. Of the 4387 patients taking VKAs before entry into the trial, 1334 were not taking warfarin (Table****2). Detailed features of patients participating in the ROCKET AF trial, stratified by geographic region, are presented in Table S1.

**Table 1.** Mean i‐TTR by Baseline Characteristics: Clinical and Demographic Features

Baseline Variable	N (%)	i‐TTR (Mean %)	Univariable *P* Value
Age, y			<0.0001
<73	3487 (50)	53.6±20.9	
≥73	3496	56.8±21.5	
Sex			<0.0001
Male	4242 (61)	56.4±21.2	
Female	2741	53.3±21.3	
Race			<0.0001
White	5829 (83)	56.3±20.9	
African American	83 (1)	51.9±21.0	
Asian	872 (12)	48.3±22.4	
American Indian/Alaskan	10	51.2±19.1	
Hawaiian/ Pacific Islander	4	60.1±12.5	
Other	185 (3)	52.6±21.7	
Region			<0.0001
East Asia	727 (10)	50.4±21.4	
India	130 (2)	35.9±23.3	
Eastern Europe	2663 (38)	49.7±21.2	
Western Europe/similar	1088 (16)	63.2±18.5	
South Africa	124 (2)	54.8±22.1	
Latin America	924 (13)	55.2±20.0	
Canada/ United States	1327 (19)	64.1±18.2	
BMI, kg/m^2^			0.0003
<28	3426 (49)	54.3±21.4	
≥28	3553	56.0±21.1	
Systolic BP, mm Hg			0.0005
<130	2675 (38)	56.8±20.8	
≥130	4300	54.2±21.5	
AF type			0.79
Persistent	5648 (81)	55.1±21.5	
Paroxysmal	1239 (18)	55.3±20.5	
New onset/ diagnosis	96 (1)	56.5±19.5	
Hypertension			0.055
Absence	649	56.7±21.1	
Presence	6334 (91)	55.0±21.3	
Diabetes			0.61
Absence	4230	55.3±21.3	
Presence	2753 (39)	55.0±21.2	
Prior stroke or TIA			0.087
Absence	3338	55.6±21.2	
Presence	3645 (52)	54.8±21.3	
Congestive heart failure			<0.0001
Absence	2642	59.0±20.7	
Presence	4340 (62)	52.9±21.2	
eGFR (MDRD),^15^ mL/min per 1.73 m^2^			0.016
<68	3431 (49)	55.3±21.2	
≥68	3548	55.0±21.4	
Baseline hemoglobin, g/L			0.0052
<10.0	2469 (35)	53.5±21.5	
≥10.0	4510	56.1±21.1	
CAD			<0.0001
Absence	5294	54.6±21.3	
Presence	1689 (24)	57.0±21.0	
COPD			0.053
Absence	6259	55.4±21.3	
Presence	719 (10)	53.7±21.1	
PAD			0.047
Absence	6558	55.1±21.3	
Presence	425 (6)	57.2±20.7	
Prior GI bleed			<0.0001
Absence	6713	55.0±21.3	
Presence	270 (4)	60.7±19.3	
Liver disease			0.012
Absence	6616	55.3±21.2	
Presence	367 (5)	52.5±21.9	
Alcohol consumption (past 12 mo)			<0.0001
Abstinent	4516 (65)	53.2±21.4	
Light	2117 (30)	58.6±20.5	
Moderate	302 (4)	62.1±19.9	
Heavy	47 (1)	48.8±22.2	
CHADS_2_ score*			<0.0001 L
1*	2	33.3±47.1	0.0015 Q
2	920 (13)	59.3±19.7	
3	3094 (44)	55.1±21.3	
4	1963 (28)	54.3±21.7	
5	852 (12)	53.6±21.3	
6	152 (2)	53.4±21.5	

Continuous predictors are split at median for summarizing TTR but tested as continuous. Missing data occurred in fewer than 0.2% of records for any variable. Race: for purposes of testing, race groups were white, Asian, and all others. i‐TTR indicates individual patient‐level time in therapeutic range; BMI, body mass index; BP, blood pressure; AF, atrial fibrillation; TIA, transient ischemic attack; eGFR, estimated glomerular filtration rate; MDRD, Modification of Diet in Renal Disease; CAD, coronary artery disease; COPD, chronic obstructive pulmonary disease; PAD, peripheral artery disease; GI, gastrointestinal.

* CHADS_2_: linear (L) and quadratic (Q) models tested.

* CHADS_2_=1: combined with CHADS_2_=2 for testing.

**Table 2.** Mean i‐TTR by Baseline Characteristics: Medication Use

Baseline Variable	N	i‐TTR Mean %	Univariable *P* Value
Prior VKA experience			<0.0001
VKA naïve	2596 (37)	47.4±22.1	
VKA experienced but warfarin naïve	1334 (19)	56.9±19.0	
Warfarin experienced	3053 (44)	61.1±19.3	
Aspirin			<0.0001
Absence	4949 (71)	56.4±20.8	
Presence	2034	52.2±22.1	
ACE inhibitor			0.070
Absence	3223	55.7±21.3	
Presence	3760 (54)	54.8±21.2	
ACE inhibitor or ARB			0.86
Absence	1808	55.3±21.4	
Presence	5175 (74)	55.2±21.2	
Amiodarone			<0.0001
Absence	6444	55.7±21.2	
Presence	539 (8)	49.3±20.6	
Digitalis			0.0037
Absence	4278	55.8±21.3	
Presence	2705 (39)	54.3±21.2	
β‐Blocker			0.0003
Absence	2411	53.9±21.8	
Presence	4572 (65)	55.8±21.0	
Loop diuretic			0.75
Absence	4577	55.1±21.3	
Presence	2406 (34)	55.3±21.3	
Statin			<0.0001
Absence	3968	52.7±21.6	
Presence	3015 (43)	58.4±20.3	

There were no missing data for this Table. i‐TTR indicates individual patient‐level time in therapeutic range; VKA, vitamin K antagonist; ACE, angiotensin‐converting enzyme; ARB, angiotensin receptor blocker.

### Univariable Associations With i‐TTR

The overall mean i‐TTR was 55.2% (SD 21.3%), and the median i‐TTR was 57.9% (interquartile range [IQR] 43.0% to 70.6%). The mean time with INR <2 was 29.1%, and the mean time with an INR >3 was 15.7%. When the definition of therapeutic range was expanded to INR 1.8 to 3.5, the mean time in this therapeutic range was 74.5% (SD 21.8%) and the median was 80.4% (IQR 65.9% to 89.9%). In univariable analysis, multiple patient features were associated with i‐TTR (Tables****1 and 2). Younger patients, female patients, those with heart failure, and patients with higher CHADS_2_ scores had lower mean i‐TTR levels. Asian patients had a mean i‐TTR a full 8% lower than did white patients. Patients who reported light to moderate alcohol consumption had higher i‐TTR levels than did those who were alcohol abstinent. Prior experience with warfarin had a notably strong association with i‐TTR. Warfarin‐experienced patients had a mean i‐TTR of 61.1% compared with a mean of 47.4% for VKA‐naïve patients. Patients who had previously taken non–warfarin VKAs had a mean i‐TTR of 56.9%. Multiple other medications taken at entry into the study were statistically associated with i‐TTR. During follow‐up, 2287 patients had ≥1 hospitalizations. There was no decrease in mean i‐TTR among these hospitalized patients compared with those avoiding hospitalization during the trial (data not shown).

### Association of Geographic Region and Mean i‐TTR

**Figure 1.**
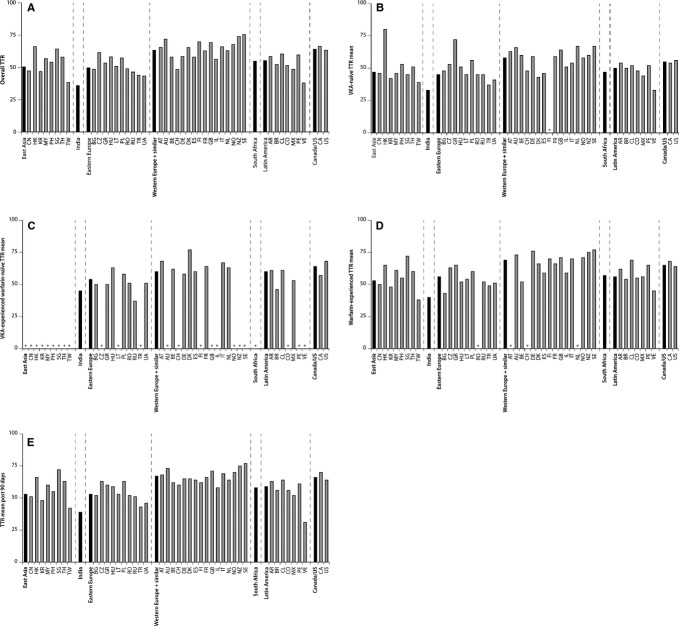
A, Overall distribution of mean i‐TTR (%) by country (gray bars), grouped by region (black bars). B, Distribution of mean i‐TTR (%) by country (gray bars), grouped by region (black bars), for patients naïve to vitamin K antagonist therapy at baseline. C, Distribution of mean i‐TTR (%) by country (gray bars), grouped by region (black bars), for patients experienced with vitamin K antagonist therapy but naïve to warfarin treatment at baseline. D, Distribution of mean i‐TTR (%) by country (gray bars), grouped by region (black bars), for warfarin‐experienced patients at baseline. E, Distribution of overall mean i‐TTR (%) by country (gray bars), grouped by region (black bars), excluding the first 90 days in the trial. TTR indicates time in therapeutic range at individual patient level; East Asia: CN, China; HK, Hong Kong; KR, Korea; MY, Malaysia; PH, Philippines; SG, Singapore; TH, Thailand; TW, Taiwan; Eastern Europe: BG, Bulgaria; CZ, Czech Republic; GR, Greece; HU, Hungary; LT, Lithuania; PL, Poland; RO, Romania; RU, Russia; TR, Turkey; UA, Ukraine; Western Europe/similar: AT, Austria; AU, Australia; BE, Belgium; CH, Switzerland; DE, Germany; DK, Denmark; ES, Spain; FI, Finland; FR, France; GB, Great Britain; IL, Israel; IT, Italy; NL, Netherlands; NO, Norway; NZ, New Zealand; SE, Sweden; Latin America: AR, Argentina; BR, Brazil; CL, Chile; CO, Colombia; MX, Mexico; PE, Peru; VE, Venezuela.

There was remarkable variation in mean i‐TTR across geographic regions, ranging from 36% in the small group of patients treated in India to 50% for patients treated in Eastern Europe and East Asia to 63% and 64%, respectively, for patients treated in Western Europe/similar and Canada/United States (Figure 1a). Across regions, there were marked differences in the proportion of patients naïve to VKAs, ranging from 9.7% of patients in Canada/United States to 53% in Eastern Europe. In Canada/United States, East Asia, and South Africa, the predominant VKA used before the trial was warfarin. But, in the remaining regions, many patients who were VKA experienced were still warfarin naïve (Table****3). Nonetheless, the qualitative regional patterns of i‐TTR across regions persisted after stratification by patients' experience with VKAs, although the absolute differences in mean TTR were modestly reduced. Patients treated in Canada/United States and Western Europe/similar still had the highest TTRs, whether patients were experienced with warfarin or completely VKA naïve. The point estimates were modestly higher for Western Europe/similar versus Canada/United States in these stratified analyses (Table****3). Country‐level patterns of mean i‐TTR stratified by warfarin experience at baseline are presented in Figure 1b through 1d.

**Table 3.** Regional Mean i‐TTR by Prior VKA Experience

	N	i‐TTR, mean %	SE	Median (25th, 75th)	Parameter Estimate	*P* Value
VKA naïve						
East Asia	356	47.3	1.1	49 (34, 63)	−7.75	0.0005
India	87	32.6	2.5	29 (13, 49)	−22.46	<0.0001
Eastern Europe	1414	45.2	0.6	47 (31, 61)	−9.90	<0.0001
Western Europe/similar	233	57.8	1.3	62 (48, 72)	2.72	0.25
South Africa	29	46.5	4.8	47 (26, 64)	−8.57	0.054
Latin America	348	50.1	1.1	54 (37, 64)	−5.00	0.025
Canada/United States	129	55.1	1.8	58 (46, 70)	Ref	
VKA experienced but warfarin naïve*						
East Asia	0					
India	20	45.5	5.1	47 (28, 63)	−14.72	0.0006
Eastern Europe	619	53.6	0.8	55 (43, 68)	−6.53	<0.0001
Western Europe/similar	399	60.1	0.9	63 (50, 73)	Ref	
South Africa	0					
Latin America	293	60.1	1.0	62 (50, 72)	−0.08	0.96
Canada/United States	3	64.1	3.8	65 (57, 70)		
Warfarin experienced						
East Asia	371	53.3	1.1	56 (41, 68)	−11.83	<0.0001
India	23	39.9	4.6	42 (27, 52)	−25.25	<0.0001
Eastern Europe	630	55.9	0.7	58 (45, 70)	−9.16	<0.0001
Western Europe/similar	456	68.7	0.7	70 (60, 79)	3.61	0.64
South Africa	95	57.3	2.1	63 (46, 71)	−7.76	<0.0001
Latin America	283	56.4	1.2	59 (45, 71)	−8.75	<0.0001
Canada/United States	1195	65.1	0.5	67 (55, 78)	Ref	

i‐TTR indicates individual patient‐level time in therapeutic range; VKA, vitamin K antagonist.

* Because there are only 3 patients in this group in North America, Western Europe is used as the reference instead. There are no patients in this group in East Asia or South Africa.

By 90 days, patients newly started on warfarin should have achieved a relatively stable dose. Analyses excluding the first 90 days of follow‐up resulted in improved regional mean i‐TTRs, particularly in regions with a high proportion of patients naïve to warfarin. Despite these changes, the regional‐ (Table****4) and country‐level (Figure****1e) differences persisted. When we further stratified these analyses starting at 90 days by patients' previous exposure to warfarin and VKAs, the regional TTR patterns were preserved (data not shown).

**Table 4.** Regional Mean i‐TTR After First 90 Days of Follow‐up

Region	N	i‐TTR, mean %	SD	Median (25th, 75th)	Parameter Estimate	*P* Value
East Asia	677	53.3	21.7	56 (40, 67)	−12.52	<0.0001
India	115	39.5	25.2	42 (21, 56)	−26.38	<0.0001
Eastern Europe	2462	53.0	21.5	55 (40, 68)	−12.82	<0.0001
Western Europe/similar	1019	66.6	17.7	69 (58, 79)	0.76	0.37
South Africa	115	57.6	21.1	59 (46, 74)	−8.19	<0.0001
Latin America	875	59.0	20.0	61 (48, 74)	−6.84	<0.0001
Canada/United States	1244	65.8	18.7	68 (56, 79)	Ref	

i‐TTR indicates individual patient‐level time in therapeutic range.

### Multivariable Associations With i‐TTR

In multiple linear regression models of mean i‐TTR, many features remained statistically significant (Table****5). Particularly strong effects were seen for patients who were VKA naïve (−9.1%, compared with warfarin experienced), women (−9.3%), patients with heart failure (−3.2%), patients with chronic obstructive pulmonary disease (−2.9%), patient using amiodarone at study entry (−3.6%), and across categories of use of alcohol. Even after adjustment for all other significant features (but leaving out race because of collinearity with region), the effect of geographic region remained remarkable. With Canada/United States patients as the referent group, the mean i‐TTR was an absolute 8.6% lower for patients in Eastern Europe, 8.7% lower in East Asia, 4.7% lower in Latin America, and essentially the same for patients treated in Western Europe/similar. The small sample of patients in India had a particularly low average i‐TTR. Although there were several strong determinants of mean i‐TTR, the overall multivariable model *R*^2^ was only 16%. By percent of variance explained (ie, partial *R*^2^), the strongest risk factors were VKA experience (3.7%) and geographic region (4.3%).

**Table 5.** Predictors of i‐TTR (%) in Warfarin Patients by Multiple Linear Regression Modeling*: Geographical Site of Recruitment Grouped as Regions

Baseline Characteristic	Multivariable			
	Parameter Estimate	*F*	*P* Value	Partial *R*^2^
VKA experience				
Warfarin experienced	Ref	129.71	<0.0001	0.0374
VKA experienced/warfarin naïve	−2.221			
VKA naïve	−9.138			
Region				
Canada/United States	Ref	49.24	<0.0001	0.0426
Western Europe/similar	0.680			
Latin America	−4.692			
South Africa	−8.347			
Eastern Europe	−8.598			
East Asia	−8.680			
India	−20.559			
CHF	−3.172	37.05	<0.0001	0.0053
Female	−9.324	22.74	<0.0001	0.0033
COPD	−2.859	13.34	0.0003	0.0019
eGFR, 10 mL/min per 1.73 m^2^				0.0030
Linear	−6.474	11.36	0.0008	
Quadratic	0.169	4.77	0.029	
Hemoglobin, 2 g/L				0.0029
Linear	−8.794	11.17	0.0008	
Quadratic	0.276	5.02	0.025	
Systolic BP	−0.0453	9.39	0.0022	0.0014
BMI				0.0022
Linear	0.677	7.38	0.0066	
Quadratic	−0.0086	5.13	0.024	
Diabetes	−1.208	5.77	0.016	0.0008
Alcohol consumption, 12 mo				
Abstinent	Ref	5.76	0.0006	0.0025
Light	1.772			
Moderate	3.012			
Heavy	−4.921			
Medications at entry to the trial				
Amiodarone	−3.608	16.42	<0.0001	0.0024
Statin	1.682	11.36	0.0008	0.0016
Aspirin	−1.111	4.10	0.043	0.0006

i‐TTR indicates individual patient‐level time in therapeutic range; VKA, vitamin K antagonist; CHF, congestive heart failure; COPD, chronic obstructive pulmonary disease; eGFR, estimated glomerular filtration rate; BP, blood pressure; BMI, body mass index.

* For the multivariable model results reported in Table 4a through 4c, n=6961 with 22 subjects omitted because of missing covariate values.

### Variation in i‐TTR Across Countries

There was substantial variation in i‐TTR across the 45 countries in ROCKET AF, ranging from a mean of 36% to 75%. Substitution of individual countries for geographic regions in the multiple linear regression model led to an increase in the overall model *R*^2^ from 16% to 19% (Table****6). Even within regions, there was considerable variability across countries (Figure****1). Of particular interest, the mean TTR was 47% in China and 38% in Taiwan but 66% in Hong Kong and 64% in Singapore. Ninety‐nine percent of the patients in all 4 of these regions were identified as being of Asian race. When we substituted patient's race for patient's region in the multivariable model, the overall model *R*^2^ deteriorated to 12.8% (Table****7).

**Table 6.** Predictors of i‐TTR (%) in Warfarin Patients by Multiple Linear Regression Modeling: Geographical Site of Recruitment Grouped as Countries

Baseline Characteristic	Multivariable			
	Parameter Estimate	*F*	*P* Value	Partial *R*^2^
VKA experience		74.94	<0.0001	0.0217
Warfarin experienced	Ref			
VKA experienced/warfarin naïve	−7.399			
VKA naïve	−2.153			
CHF	−2.373	20.19	<0.0001	0.0029
Country		13.73	<0.0001	0.0876
United States	Ref			
Argentina	−1.486			
Australia	7.531			
Austria	5.945			
Belgium	−3.014			
Brazil	−7.721			
Bulgaria	−9.669			
Canada	2.801			
Chile	−0.302			
China	−11.081			
Colombia	−8.315			
Czech Republic	−1.641			
Denmark	1.548			
Finland	4.294			
France	1.180			
Germany	−1.600			
Greece	−7.210			
Hong Kong	3.003			
Hungary	−3.170			
India	−21.495			
Israel	−4.492			
Italy	3.818			
Korea	−14.885			
Lithuania	−10.173			
Malaysia	−4.867			
Mexico	−10.713			
Netherlands	−0.160			
New Zealand	9.905			
Norway	4.564			
Peru	−2.116			
Philippines	−3.744			
Poland	−1.884			
Romania	−10.103			
Russia	−10.903			
Singapore	2.828			
South Africa	−7.806			
Spain	−2.821			
Sweden	12.009			
Switzerland	−10.068			
Taiwan	−20.772			
Thailand	−3.865			
Turkey	−16.503			
Ukraine	−13.930			
United Kingdom	6.014			
Venezuela	−20.635			
COPD	−2.669	11.91	0.0006	0.0017
Female	−6.275	10.24	0.0014	0.0015
Diabetes	−1.598	10.22	0.0014	0.0015
BMI				0.0022
Linear	0.744	9.14	0.0025	
Quadratic	−0.010	6.58	0.010	
Systolic BP	−0.037	6.28	0.012	0.0009
Hemoglobin, 2 g/L				0.0014
Linear	−4.654	3.11	0.078	
Quadratic	0.109	0.78	0.38	
eGFR, 10 mL/min per 1.73 m^2^				0.0013
Linear	−3.478	3.26	0.071	
Quadratic	0.073	0.90	0.34	
Alcohol consumption, 12 mo		2.41	0.065	0.0010
Abstinent	Ref			
Light	0.838			
Moderate	1.624			
Heavy	−5.340			
Medications at entry to the trial				
Amiodarone	−2.616	8.74	0.0031	0.0013
Statin	0.818	2.63	0.11	0.0004
Aspirin	−0.619	1.25	0.26	0.0002

i‐TTR indicates individual patient‐level time in therapeutic range; VKA, vitamin K antagonist; CHF, congestive heart failure; COPD, chronic obstructive pulmonary disease; BMI, body mass index; BP, blood pressure; eGFR, estimated glomerular filtration rate.

**Table 7.** Predictors of i‐TTR (%) in Warfarin Patients by Multiple Linear Regression Modeling Using Race in Model Rather Than Region

Baseline Characteristic	Multivariable			
	Parameter Estimate	*F*	*P* Value	Partial *R*^2^
VKA experience		239.37	<0.0001	0.0690
Warfarin experienced	Ref			
VKA experienced/warfarin naïve	−11.965			
VKA naïve	−4.149			
CHF	−4.942	92.90	<0.0001	0.0132
Female	−14.294	37.94	<0.0001	0.0054
Race		20.47	<0.0001	0.0088
White	Ref			
Black	1.924			
Asian	−5.919			
Other	−3.836			
eGFR, 10 mL/min per 1.73 m^2^				0.0048
Linear	−11.123	24.26	<0.0001	
Quadratic	0.331	14.26	0.0002	
Hemoglobin, 2 g/L				0.0043
Linear	−15.327	24.42	<0.0001	
Quadratic	0.559	15.92	<0.0001	
Systolic BP	−0.055	13.42	0.0003	0.0019
Alcohol consumption, 12 mo		10.09	<0.0001	0.0044
Abstinent	Ref			
Light	2.427			
Moderate	4.541			
Heavy	−3.944			
COPD	−2.305	8.44	0.0037	0.0012
BMI				0.0022
Linear	0.422	2.80	0.095	
Quadratic	−0.004	1.13	0.29	
Diabetes	−0.638	1.57	0.21	0.0002
Medications at entry to the trial				
Amiodarone	−4.395	23.89	<0.0001	0.0034
Statin	2.568	26.35	<0.0001	0.0038
Aspirin	−0.782	2.02	0.16	0.0003

i‐TTR indicates individual patient‐level time in therapeutic range; VKA, vitamin K antagonist; CHF, congestive heart failure; eGFR, estimated glomerular filtration rate; BP, blood pressure; COPD, chronic obstructive pulmonary disease; BMI, body mass index.

### Distribution of INR Values and Management of Test Results

**Figure 2.**
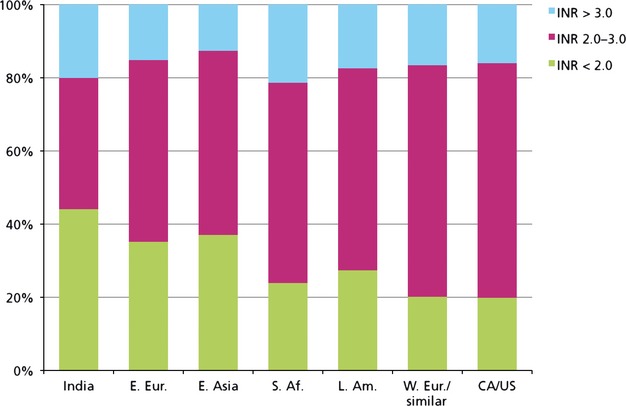
Distribution of times in therapeutic (INR 2.0 to 3.0), low, and high INR range by geographic region. INR indicates international normalized ratio.

Geographic regional variation in mean i‐TTR levels was largely due to time at INR <2.0 (Figure****2, Table****8). For Canada/United States patients, the mean time at INR <2.0 was 19.9%. In contrast, for Eastern European patients, the mean time at INR <2.0 was 35.2%, and for patients in East Asia, the time at INR <2.0 was 37.1%. The differences in mean time at INR >3.0 were much smaller and contributed much less to the differences in i‐TTR across regions. If we define “dangerously” low as INR <1.7, the regional pattern persisted with patients in Canada/United States and Western Europe/similar dangerously low ≈8% of the time compared with 19.7% of the time in Eastern Europe and 18.8% in East Asia. Canada/US and Western Europe/similar centers were in the “expanded” therapeutic range of INR 1.8 to 3.5 a mean of 84% and 83% of the time, respectively. By contrast, patients in East Asia and Eastern Europe were in this expanded range ≈70% of the time.

**Table 8.** i‐TTR, Time in Other INR Ranges, and INR Test Results and Frequency by Region

Parameter	Region								
	Statistic	Total	East Asia	India	EasternEurope	Western Europe/similar	South Africa	Latin America	Canada/United States
i‐TTR									
i‐TTR (INR 2.0 to 3.0, %)	N (patients)	6983	727	130	2663	1088	124	924	1327
	Mean±SD	55.2±21.27	50.4±21.42	35.9±23.34	49.7±21.16	63.2±18.48	54.8±22.011	55.2±20.00	64.1±18.23
	(25th, 75th percentile)	(43.0, 70.6)	(37.6, 64.8)	(18.6, 50.7)	(36.8, 64.8)	(53.7, 75.9)	(42.9, 70.6)	(44.5, 69.3)	(54.8, 77.0)
Time above TR (INR >3.0, %)	N	6983	727	130	2663	1088	124	924	1327
	Mean±SD	15.7±13.13	12.6±12.83	20.0±21.34	15.1±12.61	16.5±13.20	21.3±18.10	17.4±12.95	16.0±12.28
	(25th, 75th percentile)	(7.0, 21.5)	(3.7, 17.0)	(6.1, 25.5)	(6.2, 21.2)	(7.4, 22.7)	(8.7, 28.3)	(9.0, 23.5)	(8.3, 21.1)
Time below TR (INR <2.0, %)	N	6983	727	130	2663	1088	124	924	1327
	Mean±SD	29.1±21.94	37.1±23.00	44.1±25.87	35.2±22.78	20.2±17.88	23.9±23.25	27.4±20.01	19.9±16.16
	(25th, 75th percentile)	(13.6, 38.6)	(20.6, 51.0)	(25.2, 61.0)	(19.2, 46.3)	(8.7, 26.2)	(7.3, 32.9)	(14.2, 34.1)	(9.1, 26.7)
Time dangerously out of range—low (INR <1.7)	N	6983	727	130	2663	1088	124	924	1327
	Mean±SD	14.8±19.10	18.8±19.92	30.9±25.73	19.7±21.58	8.0±13.19	11.9±18.61	13.8±17.77	7.7±11.74
	(25th, 75th percentile)	(2.7, 18.7)	(5.6, 27.0)	(13.6, 48.0)	(5.2, 26.1)	(1.1, 9.5)	(1.1, 14.2)	(3.2, 15.9)	(1.2, 9.7)
Time in INR range of 1.8 to 3.5	N	6983	727	130	2663	1088	124	924	1327
	Mean±SD	74.5±21.75	69.9±22.37	51.8±27.30	68.8±22.90	82.7±17.19	75.6±23.62	74.8±20.36	83.6±15.64
	(25th, 75th percentile)	(65.9, 89.9)	(59.2, 85.9)	(33.1, 72.2)	(58.6, 85.7)	(77.1, 94.1)	(69.9, 90.8)	(67.8, 88.4)	(78.2, 93.8)
INR tests (multiple observations/patient)	N (tests)	181 640	17 218	2415	64 644	30 439	3131	22 134	41 659
Average INR
	Mean±SD	2.4±0.87	2.3±0.83	2.4±1.21	2.4±0.93	2.5±0.80	2.5±0.86	2.4±0.92	2.5±0.78
	Median	2.3	2.2	2.1	2.2	2.4	2.4	2.3	2.4
	(25th, 75th percentile)	(1.8, 2.8)	(1.7, 2.7)	(1.5, 2.8)	(1.7, 2.8)	(2.0, 2.9)	(1.9, 2.9)	(1.8, 2.8)	(2.0, 2.9)
Average No. of tests/patient	Mean±SD	26±13.3	24±10.8	19±11.5	24±11.6	28±13.9	25±10.9	24±10.6	31±17.0
	(25th, 75th percentile)	(18, 34)	(17, 32)	(8, 27)	(17, 32)	(19, 37)	(19, 33)	(17, 31)	(19, 43)
Average No. of days between INR measurements	N	6983	727	130	2663	1088	124	924	1327
	Mean±SD	21±5.1	23±4.7	22±5.6	23±5.0	20±5.3	21±4.8	22±4.3	19±4.8
	(25th, 75th percentile)	(19, 25)	(22, 26)	(20, 26)	(21, 26)	(16, 24)	(19, 24)	(20, 25)	(17, 23)

i‐TTR indicates individual patient‐level time in therapeutic range; INR, international normalized ratio.

The distributions of INR values confirmed that lower i‐TTRs resulted primarily from subtherapeutic INR levels (Table****8). The median INR was 2.4 for patients in Canada/United States and Western Europe/similar but 2.2 for patients in Eastern Europe and East Asia and 2.3 for patients in Latin America. The distributions were narrower in Canada/United States and Western Europe/similar with IQRs of 0.9 INR unit compared with East Asia and Latin America with IQRs of 1.0 INR unit and Eastern Europe with an IQR of 1.1 INR units (all *P<*0.001)

**Figure 3.**
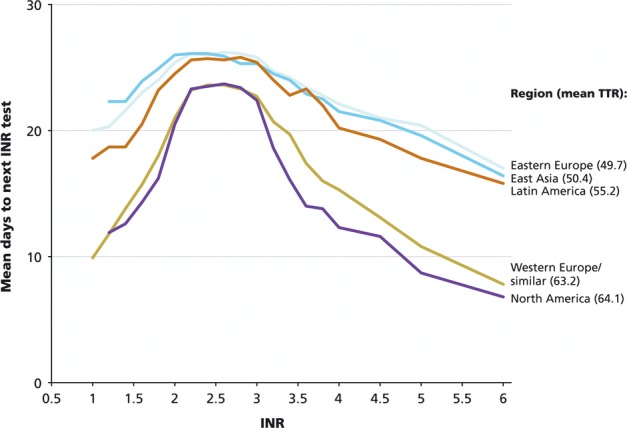
Inter‐INR test interval by value of first INR, stratified by geographic region. INR indicates international normalized ratio; TTR, time in therapeutic range.

We compared the average number of days between INR measurements (Figure****3, Table****8). Patients in Canada/United States and Western Europe had the most frequent INR tests at an average interval of 19 and 20 days, respectively. By contrast, patients in Eastern Europe and in East Asia had the least frequent INR testing with an average interval of 23 days (*P<*0.001). We extended this analysis to compare the time to subsequent INR after an extreme INR value. There was marked variation in median time to a follow‐up INR test after an INR ≤1.5, from 9 days in Western Europe/similar and Canada/United States to 26 and 27 days in Eastern Europe and East Asia, respectively (*P<*0.001). Similarly, the median time to a subsequent INR test after an INR ≥4.0 was 7 days in Western Europe/similar and Canada/United States versus 25 and 23 days in Eastern Europe and East Asia, respectively (*P<*0.001). Figure****3 displays the distribution of mean inter‐test intervals as a function of initial INR test result. There is an inverted “U” pattern seen in all regions but inter‐test intervals were shortest in regions with the highest TTRs, regardless of initial test result.

## Discussion

The linearly interpolated TTR has become a widely accepted measure of the quality of anticoagulation management.^7^ In the ROCKET AF trial, the mean i‐TTR for patients assigned to warfarin was 55%, lower than that reported for other recent trials of novel anticoagulants,^17–19^ with a wide range of i‐TTR values. The ROCKET AF trial enrolled patients at particularly high risk of stroke. One of these stroke risk factors, heart failure, was associated with an adjusted 3% decrease in average i‐TTR. However, other standard stroke risk factors in AF (ie, prior stroke/TIA, hypertension, and diabetes) had only modest, if any, univariate effects on i‐TTR and none was selected as a significant predictor of i‐TTR in our multivariable model. The 2 strongest determinants of i‐TTR (by partial *R*^2^) were not patient comorbidities but rather pretrial experience with warfarin and geographic site. Patients who were taking warfarin before entry into the trial had an adjusted absolute 9% higher mean i‐TTR than those who were VKA naïve. Interregional mean i‐TTR varied by as much as 21%, even after accounting for patient clinical features and experience with warfarin. Excluding the small number of patients treated in India, interregional differences in mean i‐TTR still spanned an absolute 8.7%. Excluding the first 90 days of warfarin treatment, to allow warfarin‐naïve patients to become warfarin‐experienced, still resulted in large interregional differences in mean TTR. When country was included in the model instead of region, the total variance explained increased. Large regional effects on TTR with similar geographic patterns have been observed in other recent trials.^8,20^

Poorer i‐TTR primarily reflected INR values below 2.0. The risk of stroke among patients with AF rises steeply the lower the INR value below 2.0.^1^ The TTR does not distinguish low from very low INR values. When we assessed time in dangerously low INR range (ie, INR <1.7) the regional differences persisted. When we focused specifically on the regional distributions of INR values, we found the lower the mean i‐TTR, the lower the mean INR. For East Asia, in particular, the IQR of INR values was nearly as narrow as that for the regions with higher TTRs, suggesting that physicians were implicitly targeting the low end of the target range of INR 2.0 to 3.0. The fact that the mean TTR for patients treated in Hong Kong and Singapore was much higher than the mean TTR in the remaining countries in East Asia indicates that medical care practices and not race determine anticoagulation control in this region.^21^ We found, as well, that regions with lower TTR values obtained INR tests less frequently. While follow‐up test intervals were shorter the more extreme the initial INR results, the intervals were still longer in regions with the lowest TTR. Rose et al have proposed as a quality indicator the time to follow‐up INR after an INR ≤1.5 or ≥4.0.^22^ We found longer test intervals after such extreme INR values in regions with lower mean TTRs.

We have used the distinctively large and geographically diverse cohort of warfarin‐treated patients in the ROCKET AF trial to add to our understanding of the determinants of TTR. Our study benefits from the high quality of information captured at study entry, close longitudinal follow‐up, and standardized INR testing with a single type of point‐of‐care device. There was comprehensive recording of clinically scheduled INR test results. As a consequence of the trial protocol, patients did not go for long periods (ie, >8 weeks) without an INR test, consistent with guideline recommendations.^14^ This is in contrast to usual clinical care where the distribution of test intervals is broader, adding uncertainty to the calculation of TTR.^23^

Our study has limitations. As always, our trial‐based results may not generalize completely to usual clinical care. Our regional findings reflect the care provided by a limited set of investigators in any geographic region. As such, we cannot claim that our findings are clearly representative of warfarin management in participating countries. However, it is notable that the regional pattern of our results is similar to those reported by the ACTIVE W trial for the countries where both trials recruited patients.^8^ In particular, in both the ROCKET AF and ACTIVE W trials the country with the highest i‐TTR was Sweden and a recent survey of anticoagulation in clinical care in Sweden reported a mean i‐TTR of 74%, nearly identical to the value of 75% observed in the ROCKET AF trial.^24^ We cannot account for the resource constraints or cultural norms underlying different regional practices in managing warfarin. The widely used Rosendaal measurement of i‐TTR uses linear interpolation to impute daily INR values. Other imputation approaches might reduce interregional differences in i‐TTR. Ultimately, while i‐TTR makes sense as a measure of the quality of warfarin management, its quantitative relationship to the net benefit of warfarin therapy is still uncertain.

In conclusion, we found that patient clinical features, such as heart failure, were significant but modest determinants of i‐TTR. Further, we were able to quantify the reduced control of warfarin anticoagulation faced by new users of the drug. However, our most notable finding was the striking influence of geographic region, presumably reflecting different levels of aggressiveness in achieving the INR point target of 2.5, different support systems to manage warfarin, and different regional barriers to frequent INR testing and warfarin dose changes. Such geographic variation in medical practice has become a truism of health care epidemiology.^25–26^ While our understanding of the determinants of i‐TTR remains incomplete, it is clear that the providers of care, and the systems within which they work, have a profound effect on the quality of anticoagulation.
